# *β*-Hydroxy-*β*-methyl butyrate (HMB) supplementation elevates testosterone levels without significant changes to cortisol, IGF-1, or growth hormone in adults: a GRADE-assessed systematic review and meta-analysis of controlled trials

**DOI:** 10.3389/fnut.2025.1582135

**Published:** 2025-06-19

**Authors:** Mohammad Vesal Bideshki, Behrad Sadeghi, Mehrdad Behzadi, Hannane Jozi, Hadi Eskandari Damaneh, Ali Rashidinejad

**Affiliations:** ^1^Bio Environmental Health Hazards Research Center, Jiroft University of Medical Sciences, Jiroft, Iran; ^2^Student Research Committee, Jiroft University of Medical Sciences, Jiroft, Iran; ^3^Department of Agricultural, Forest and Food Science (DISAFA), University of Torino, Grugliasco, Torino, Italy; ^4^Student Research Committee, School of Nutrition and Food Sciences, Shiraz University of Medical Sciences, Shiraz, Iran; ^5^Student Research Committee, Tabriz University of Medical Sciences, Tabriz, Iran; ^6^Riddet Institute, Massey University, Palmerston North, New Zealand

**Keywords:** HMB supplementation, hormonal response, anabolic effects, testosterone, metaanalysis

## Abstract

**Background and aim:**

Increasing interest in improving physical performance and muscle mass in adults has highlighted the potential benefits of *β*-hydroxy-β-methyl butyrate (HMB) supplementation. While numerous studies have been conducted in this area, the hormonal response to HMB remains unclear. We hypothesized that HMB supplementation would significantly increase anabolic hormone levels, particularly testosterone, while not affecting the cortisol, IGF-1, or growth hormone levels in adults.

**Methods:**

A comprehensive search of databases, including PubMed, Web of Science, and Scopus, was performed to identify relevant studies until January 2024. The protocol was registered with Prospero (CRD42024552074). The studies evaluated the impact of HMB supplementation on hormonal outcomes, including testosterone, cortisol, insulin-like growth factor-1 (IGF-1), and growth hormone (GH). Utilizing a random-effects model, the standardized mean differences (SMDs) and their corresponding 95% confidence intervals (CIs) were computed, and the GRADE framework was applied.

**Results:**

A total of 15 controlled trials (CTs) comprising 712 participants were included. HMB supplementation significantly increased testosterone levels (SMD: 0.82, 95% CI: 0.35, 1.29, *p* = 0.001). However, no significant changes were observed in the cortisol (SMD: −0.39, 95% CI: −0.92, 0.14, *p* = 0.14), IGF-1 (SMD: −0.18, 95% CI: −0.54, 0.18, *p* = 0.33), and GH (SMD: 0.04, 95% CI: −0.73, 0.82, *p* = 0.91) levels. According to the GRADE criteria, the quality of evidence was rated as ranging from low to high.

**Conclusion:**

HMB supplementation significantly elevates testosterone levels in adults without distinct impacts on other hormonal pathways. However, it does not appear to significantly influence the cortisol, IGF-1, or GH levels.

## 1 Introduction

*β*-hydroxy β-methylbutyrate (HMB), a derivative of leucine breakdown, has attracted attention as a sports supplement due to its potential ergogenic properties, particularly in augmenting muscle mass and strength when combined with exercise training (1). Clinical research conducted on athletes indicates that HMB supplementation can effectively mitigate an increase in serum creatine phosphokinase levels, thereby reducing skeletal muscle damage (2–4). Moreover, HMB has been linked to improvements in strength (2, 4) and lean body mass (4), making it a favored choice among athletes and resistance trainers. HMB demonstrates positive outcomes among older adults. Lin et al. (5), in a meta-analysis, reported enhanced muscle strength in this demographic population (5). Kinoshita et al. (6) investigated 34 senior participants (aged ≥65 years) with low physical function and demonstrated significant improvements in grip strength and muscle strength through HMB supplementation for 8 weeks, without any additional exercise (6).

The mechanisms of action of HMB are commonly attributed to stabilizing the sarcolemma, a process known as the cholesterol synthesis hypothesis (CSH), and inhibiting the proteolytic pathways, typically via the ubiquitin–proteasome-dependent pathway (Ub-pathway) (7). The pre-clinical evidence indicates that HMB enhances hippocampal synaptic plasticity and restores synaptic protein levels and CREB phosphorylation in Alzheimer’s disease models, as well as promotes the differentiation of oligodendrocyte precursors for myelin repair (8–10). Studies investigating the effects of HMB on catabolic and anabolic hormonal profiles have yielded mixed results. While some studies have suggested that HMB may mitigate cortisol elevation (4) and elevate testosterone (11), others have reported that HMB has a minimal impact (12–14). Another proposed mechanism of action for HMB is through stimulation of the growth hormone (GH)/insulin-like growth factor-1 (IGF-1) axis (15).

Given its strong anabolic effect on skeletal muscle and role in muscle fiber hypertrophy (16), the potential of HMB to increase the IGF-1 concentration is significant. Hormonal adaptations, such as increases in testosterone, GH, and IGF-1, are essential for improving strength and power performance following resistance training (17, 18). Muscle loss and strength decline are common conditions among older adults, which can worsen disease progression and impede recovery. Hence, it is crucial to determine whether HMB supplementation positively impacts muscle mass. Nonetheless, conflicting evidence exists regarding GH and IGF-1. Some randomized clinical trials (RCTs) (11, 19, 20) observed increases in IGF-1 and GH with HMB supplementation, while other clinical trials found no significant effects (13, 21).

Discrepancies in the literature regarding the effects of HMB on hormones, coupled with its widespread use as an ergogenic aid, necessitate a systematic review and meta-analysis. This study aimed to systematically review and analyze the effects of HMB supplementation on testosterone, cortisol, IGF-1, and GH levels in adults. We hypothesized that HMB supplementation would significantly increase testosterone levels while having no substantial impact on cortisol, IGF-1, or GH levels. This analysis seeks to clarify the hormonal effects of HMB, potentially informing its application in improving muscle performance and health across various populations.

While existing reviews have primarily focused on athletes (22, 23), our analysis significantly broadens this scope by incorporating additional clinical trials (11, 13, 20, 24, 25) and extending to more diverse populations, such as older adults and sedentary individuals. Previous meta-analyses have included acute-phase studies with short supplementation durations (26). In contrast, our analysis is unique in that it is restricted to studies with chronic supplementation protocols, enabling us to isolate and examine the long-term effects of HMB. This comprehensive analysis fills a critical gap in the literature. It will enable the confident application of HMB supplementation across various populations, including athletes and elderly individuals (in the form of dietary supplements or incorporated into functional food/beverage products), to enhance health outcomes and optimize resistance exercise performance. This analysis represents a significant advancement over previous reviews.

## 2 Methods

We used the Preferred Reporting Items of Systematic Reviews and Meta-Analysis (PRISMA) statement guidelines to present the results of the systematic review and meta-analysis (27). Furthermore, we registered the study protocol at Prospero (CRD42024552074).

### 2.1 Search strategy

A complete search of international databases, including PubMed, Scopus, and Web of Science, was conducted from inception until January 2024 to identify relevant English-language RCTs investigating the effects of HMB supplements on hormonal responses. This research used keywords from the medical subject heading terms (MeSH) and non-MeSH terms such as “beta-hydroxy beta-methylbutyrate,” “hydroxy methylbutyrate,” “beta hydroxy beta methylbutyric acid,” “β-hydroxy β-methylbutyrate,” “leucine metabolite,” “3-hydroxyisovaleric acid,” “HMB,” “hormone,” “hormonal adaptations,” “IGF-1,” “testosterone,” “growth hormone,” and “cortisol.” Supplementary Table S1 provides comprehensive information about our search strategy. In addition to checking reference lists of relevant studies and conducting manual searches across various databases and Google Scholar, we also set up email alerts for new publications from the aforementioned databases. This comprehensive approach enabled us to stay up to date with the latest research in this field.

### 2.2 Study selection

Studies using the following Population, Intervention, Comparison, Outcomes and Study (PICOS) design criteria were selected according to the inclusion criteria (Table 1): (1) all types of RCTs, (2) all studies using any type of HMB supplementation, such as HMB, HMB-Ca, and HMB-FA, (3) having at least 2 weeks of supplementation, (4) inclusion of data on how HMB supplementation affects hormonal responses in the article, and (5) participants aged 18 years or older.

**Table 1:** Population, intervention, comparison, outcome, study (PICOS) design criteria for the inclusion and exclusion of studies.

Parameter	Criteria
Participants	Adults
Intervention	β-hydroxy-β-methylbutyrate
Comparator	Placebo
Outcomes	Possible improvements in cortisol, testosterone, insulin-like growth factor-1, and growth hormone
Study design	Clinical trial

Moreover, studies were excluded based on the following exclusion criteria: (1) all cellular, animal, and observational studies, (2) investigations lacking complete data regarding HMB supplementation and hormonal feedback, (3) studies involving participants under 18 years of age (if any), and (4) studies with a duration of less than 2 weeks. In addition, two independent individuals (HJ and BS) examined the inclusion and exclusion criteria separately, discussed the differences with another researcher (MVB), and resolved the conflicts (if any).

### 2.3 Data extraction

The screening and extraction of all data were carried out by two researchers (HJ and BS). Another researcher (MVB) was responsible for resolving any conflicts. Furthermore, an email was sent to the corresponding authors of unavailable articles to request access to the full text. Required data includes publication time; design, length, and place of the study; the first author’s name; a sample size of the intervention/placebo group; dose and type of HMB supplementation; mean and standard deviation (SD) of IGF-1, cortisol, testosterone, and GH of the intervention/placebo group before and after the study; and details of demographic information such as sex, average age, health condition, and body mass index (BMI) of subjects at the beginning of the study (extracted from the Excel-based form). The hormone units were adjusted to the most commonly used units during the data extraction. Additional information, including investigations on co-supplementation, was also gathered.

### 2.4 Quality and certainty assessment

The process of quality assessment of studies was accomplished by two researchers (HJ and BS), using the Cochrane Collaboration quality assessment tool, which consists of random sequence generation, allocation concealment, selective reporting, incomplete outcome data, personnel, participants, and assessor blinding of the research (28). A “low risk” score was assigned for fields with no defects, while a “high risk” score was assigned for fields with any defects. In addition, where the information was incomplete, an “unclear risk” score was given. Equally, the Grading of Recommendations Assessment, Development, and Evaluation (GRADE) method, including the risk of bias, precision, directness, the potential for publication bias, and consistency of results, was used to evaluate the quality of the data (29). The evidence of this approach is classified into four types: very low, low, moderate, and high.

### 2.5 Statistical analysis

The investigation of IGF-1 (ng/mL), cortisol (mcg/dL), testosterone (ng/dL), and GH (ng/mL) was conducted after HMB supplementation. The effect size was presented using weighted mean differences (WMDs), standardized mean differences (SMDs), and 95% confidence intervals (CIs). For the extraction of mean and SD values of blood hormones in both the HMB and placebo groups before and after supplementation, we calculated the mean differences and SDs by converting them using the following equation (30):


(1) $$SD^{2}=[(\mathrm{pre}\;SD^{2}+\text{post}\;SD^{2})-(2\times 0.8\times \mathrm{pre}\;\mathrm{SD}\times \text{post}\;\mathrm{SD})]$$

We calculated SDs in studies where SD was not available, and the standard error of mean (SEM) was described by calculating:


(2) SDs=SEs×square root(n)

where n refers to the number of people in the HMB/placebo group.

In addition, we converted medians, ranges, and 95% CIs to mean and SD following the method reported by Hozo et al. (31). The graphic information was also extracted using the Get Data Graph Digitizer software (32). To estimate heterogeneity, both Cochran’s Q-test and I^2^ tests were performed (*p* < 0.1 for the Q-test and I^2^ ≥ 50% for the I^2^ test) (33). The random-effects model and fixed effects model were used separately depending on the presence or absence of heterogeneity data. A sensitivity analysis was used to determine how each study affected the overall effect size by eliminating one trial at a time. A predefined subgroup analysis evaluated the effects of various factors such as type, dose, and duration of HMB supplementation, BMI, health status, age, sex, and study location on hormone status. Begg’s rank correlation and Egger’s weighted regression tests were combined with funnel plots to detect potential publication bias. The “trim and fill” and “fail-safe N” methods of Duval and Tweedie were used for publication bias analysis (34). The STATA V13 software was used to carry out this meta-analysis. A probability value (*p*-value) of less than 0.05 was deemed to indicate statistical significance.

## 3 Results

### 3.1 Flow and characteristics of the included studies

**Figure 1:**
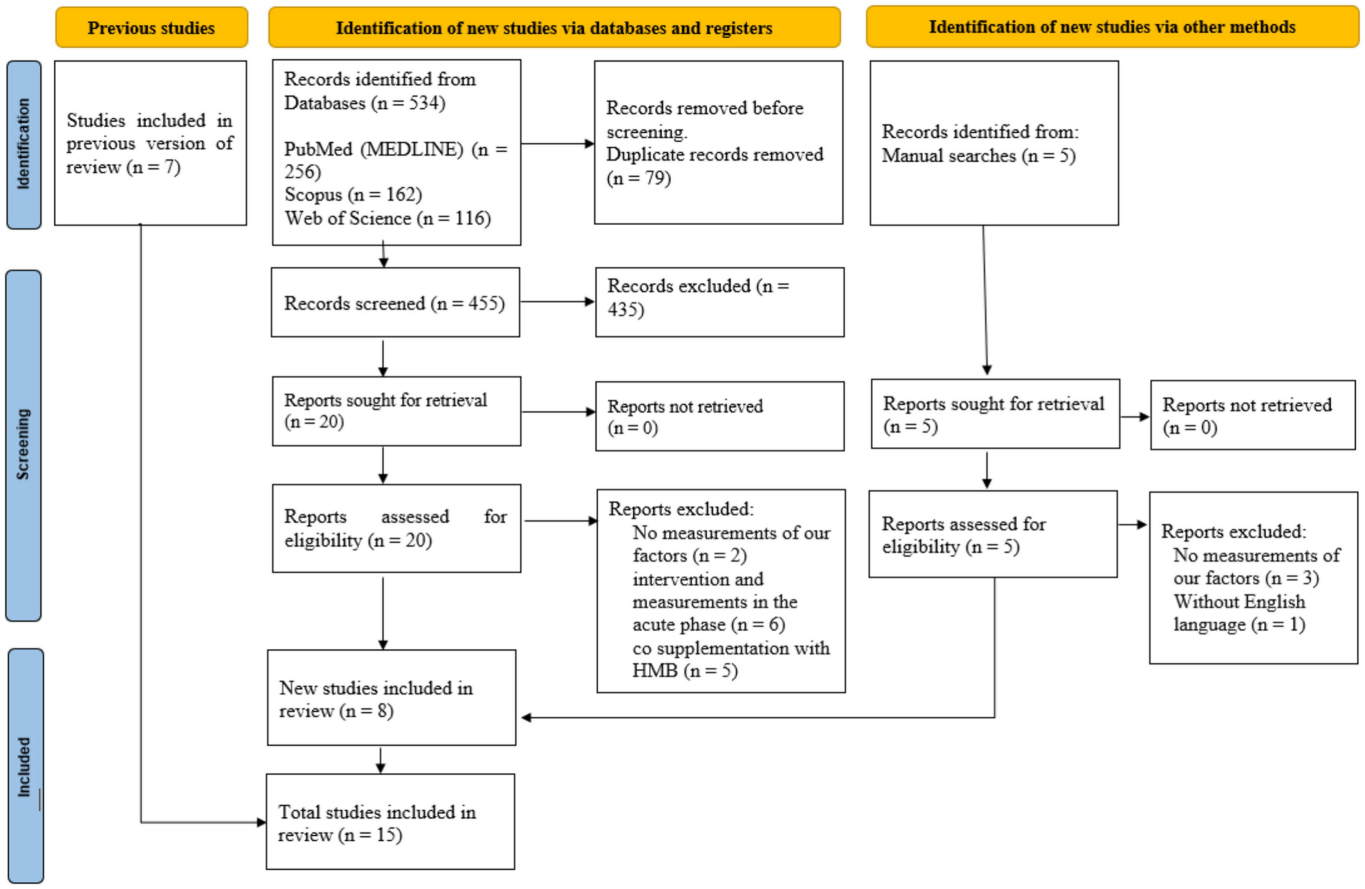
Preferred Reporting Items of Systematic Reviews and Meta-Analysis (PRISMA) 2020 flow diagram of databases searches, registers, and other sources.

In total, we identified 534 articles. After removing duplicates (*n* = 79), we retained 455 articles. A detailed review of their titles and abstracts led to the exclusion of 435 articles, as they did not meet our inclusion criteria. Consequently, 20 full-text articles were selected for further review and evaluation. Upon analysis of the full texts of these 20 studies, 12 were removed from the review due to insufficient data reporting. Thus, during this meta-analysis, we included 8 studies with 18 treatment arms. Additionally, we incorporated five studies and seven articles obtained through a manual search and from previous studies, respectively. In the final step, we included 15 CTs that met the inclusion criteria in the analysis (Figure 1).

### 3.2 Characteristics of the included studies

As shown in Table 2, the eligible studies exhibited the following characteristics. The age of the participants ranged from 19.5 (35) to 77.5 (36) years, with a mean age of 18 ≤ years. The included publications were conducted between 2003 (37) and 2023 (38). Of the 15 included studies, 3 were conducted in the USA (4, 20, 36) and Poland (12, 35, 39); 2 in Iran (19, 40) and Spain (41, 42); and 1 in Australia (37), Georgia (25), Portugal (13), Japan (43), and Switzerland (38). The majority of the studies were conducted on men, except for three studies that were conducted on both sexes (36, 38, 42) and one study that was conducted on women (43). There was a variation in trial duration from 4 (20, 38, 40, and) to 12 (4, 12, 35, 36, 39, 42, 43) weeks. Five trials (12, 25, 35, 39, 40) used a cross-over design, and the remaining 10 were conducted on a parallel basis. The trials were conducted on patients in ventilated intensive care units (38), those with bronchiectasis (42), and those with sarcopenia (36), as well as healthy (13, 19) and older (43) athletes (4, 12, 20, 25, 35, 37, 39–41).

**Table 2:** Characteristic of included studies in meta-analysis.

Study	Country	Study design	Participants	Sex	Sample size		Trial duration (Week)	Means age		Means BMI		Intervention		
					IG	CG		IG	CG	IG	CG	Type	Dose (mg/day)	Control group
Pantet et al. (38)	Switzerland	Ct, R, Db	Ventilated ICU patients with functional GI tract	Both	14	12	4 weeks	67.53	71.5	27.6	29.8	HMB-Ca	3,000	P
Pereira et al. (36)	Europe and North America	Ct, R, Db	Older adults with sarcopenia with/at-risk of malnutrition	Both	90	103	12 weeks	77.5	76	26.7 ± 4.37	26.3 ± 3.92	HMB-Ca	3,000	P
Osuka et al. (43)	Japan	Ct, R, Db	Older woman	Female	36	35	12 weeks	71.5	71.6	20.1 ± 2.2	20.7 ± 2.2	HMB-Ca + Education	1,500	P + Education
Osuka et al. (43)	Japan	Ct, R, Db	Older woman	Female	36	37	12 weeks	73.5	71.8	21.3 ± 2.2	20.9 ± 2.1	HMB-Ca + Exercise	1,500	P + Exercise
Mangine et al. (25)	Georgia	Co, R, Db	Collegiate rugby players	Male	32	32	6 weeks	20.9	21.4	NR	NR	HMB + Cr	5,000	P + Cr
Fernández-Landa et al. (41)	Spain	Ct, R, Db	Endurance Athletes	Male	7	7	10 weeks	30.43	NR	NR	NR	HMB-Ca + CrM	3,000	P + CrM
Fernández-Landa et al. (51)	Spain	Ct, R, Db	Endurance Athletes	Male	7	7	10 weeks	30.43	NR	NR	NR	HMB-Ca	3,000	P
Teixeira et al. (13)	Portugal	Ct, R, Db	Healthy men	Male	9	10	8 weeks	34	31	NR	NR	HMB-Ca + Resistance training	3,000	P + Resistance training
Teixeira et al. (13)	Portugal	Ct, R, Db	Healthy men	Male	11	10	8 weeks	30	31	NR	NR	HMB-FA + Resistance training	3,000	P + Resistance training
Asadi et al. (19)	Iran	Ct, R, Db	Healthy men	Male	8	8	6 weeks	21.5	21.3	23.9 ± 3.2	24.4 ± 4	HMB-FA	3,000	P
Durkalec-Michalski et al. (12)	Poland	Co, R, Db	Combat sports	Male	42	42	12 weeks	22.8	22.8	NR	NR	HMB-Ca	3,000	P
Durkalec-Michalski and Jeszka et al. (39)	Poland	Co, R, Db	Healthy highly trained men	Male	58	58	12 weeks	22	22	NR	NR	HMB-Ca	3,000	P
Durkalec-Michalski and Jeszka (35)	Poland	Co, R, Db	Elite male rowers	Male	16	16	12 weeks	19.5	19.5	NR	NR	HMB-Ca	3,000	NR
Townsend et al. (20)	USA	Ct, R, Db	Men without training	Male	10	10	4 weeks	23.8	21.7	NR	NR	HMB-FA + Resistance training	1,000	P + Resistance training
Arazi et al. (40)	Iran	Co, R, Db	Amateur male athletes	Male	20	20	4 weeks	22.4	22.7	24.1 ± 1.9	23.6 ± 1.5	HMB-Ca + Resistance training	3,000	P + Resistance training
Olveira et al. (42)	Spain	Ct, R	Non-cystic fibrosis patients with bronchiectasis	Male	15	15	12 weeks	58.4	53.7	25.9 ± 12.9	27.3 ± 5.8	HMB + Exercise	1,500	P + Exercise
Wilson et al. (4)	USA	Ct, R, Db	Healthy male athletes	Male	11	9	12 weeks	21.6 ± 0.5	21.6 ± 0.5	NR	NR	HMB-FA + Resistance training	3,000	P + Resistance training
Crowe et al. (37)	Australia	Ct	National Rugby League players	Male	11	6	6 weeks	24.9	24.9	NR	NR	HMB-Ca	3,000	NR

IG, intervention group; CG, control group; Pa, parallel; Db, double-blinded; Ct, controlled-trial; Co, cross over; R, randomized; placebo, P; NR, not reported; HMB, β-hydroxy-β-methylbutyrate; BCAA, branched-chain amino acid; Cr, creatine; CrM, creatine monohydrate.

### 3.3 Data quality

A summary of the results of Cochrane’s risk of bias tool for evaluating the quality of studies is presented in Table 3. The majority of studies were rated as high quality (with high bias risk in <2 domains). Conversely, only two studies were identified as low quality (with high bias risk in >2 domains) (37, 42). Using GRADE (Supplementary Table S2), evidence was categorized with varying levels of certainty. In the grading system, testosterone was assigned a high grade. This finding suggests that the estimated effects are considered accurate, and it is unlikely that further research will alter our confidence in this estimate. Both IGF-1 and GH were given a moderate grade. Finally, cortisol was assigned a very low grade, indicating that the evidence is limited and there is a significant level of uncertainty regarding the estimates of its effects.

**Table 3:** Risk of bias assessment used in this study.

Study	Random sequence generation	Allocation concealment	Selective reporting	Other sources of bias	Blinding (participants and personnel)	Blinding (outcome assessment)	Incomplete outcome data	General risk of bias
Pantet et al. (38)	L	U	L	U	L	L	L	Low
Pereira et al. (36)	L	L	L	U	L	U	L	Low
Osuka et al. (43)	L	L	L	U	L	U	L	Low
Mangine et al. (25)	L	L	U	L	L	U	L	Low
Fernández-Landa et al. (41)	L	L	L	L	L	U	L	Low
Teixeira et al. (13)	L	L	U	L	L	U	L	Low
Asadi et al. (2017)	L	U	L	U	L	U	L	Low
Durkalec-Michalski et al. (12)	L	L	L	L	L	U	L	Low
Durkalec-Michalski and Jeszka et al. (39)	L	L	L	L	L	U	L	Low
Durkalec-Michalski and Jeszka (35)	L	L	L	L	L	U	L	Low
Townsend et al. (20)	L	U	L	U	L	U	L	Low
Arazi et al. (40)	L	L	L	U	L	U	L	Low
Olveira et al. (42)	L	H	L	L	H	H	L	High
Wilson et al. (4)	L	L	U	U	L	L	L	Low
Crowe et al. (37)	H	H	L	U	H	H	L	High

L, low risk of bias; H, high risk of bias; U, unclear risk of bias.

### 3.4 Meta-analysis

#### 3.4.1 Findings from the meta-analysis of HMB and cortisol levels

**Figure 2:**
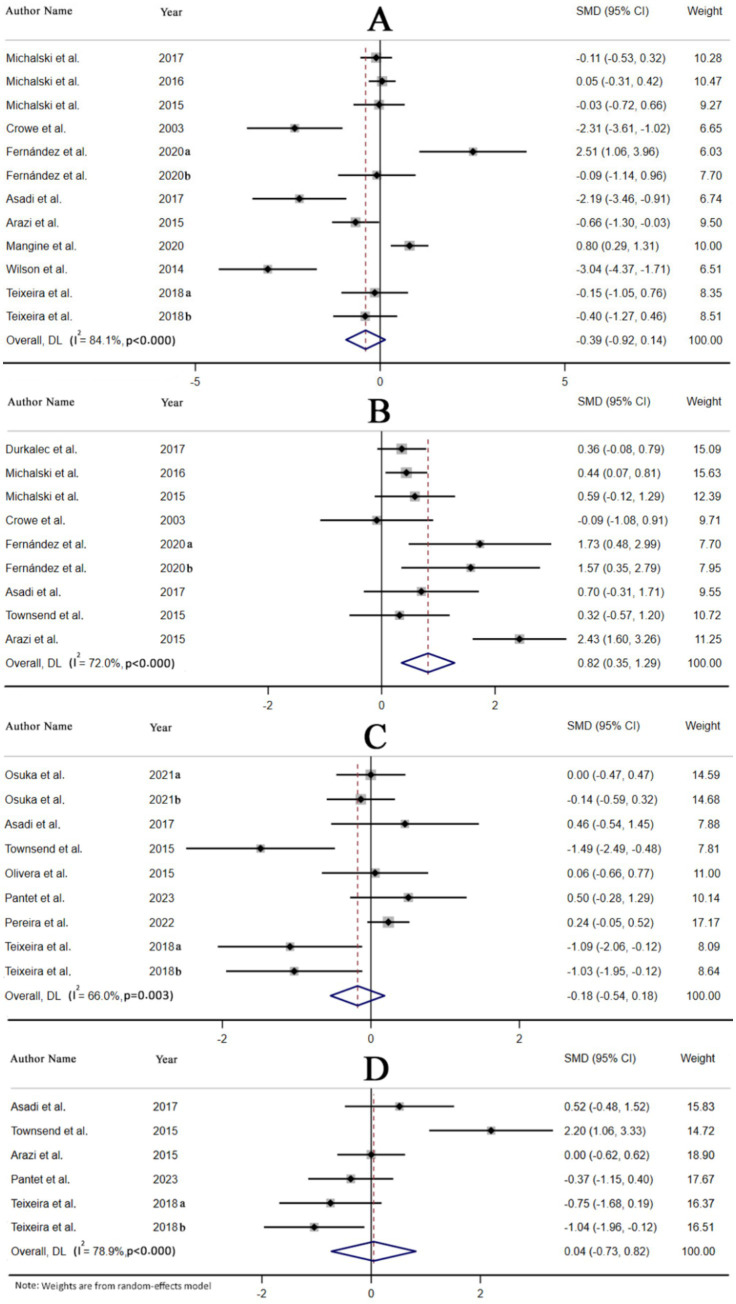
Forest plot for the effect of HMB supplementation on **(A)** cortisol, **(B)** testosterone, **(C)** insulin-like growth factor 1, and **(D)** growth hormone in adults, expressed as mean differences between intervention and control groups.

Cortisol levels were assessed in 10 studies, encompassing 12 treatment arms. Based on the pooled estimates from the random-effects model, HMB supplementation did not significantly affect cortisol reduction (SMD: -0.39, 95% CI: −0.92, 0.14, *p* = 0.14). Notably, there was significant heterogeneity among the studies (I^2^ = 84.1%, *p* < 0.001) (Figure 2A). The subgroup analysis revealed that the type of intervention used in HMBs and the location of studies could be potential contributors to this heterogeneity. Moreover, HMB supplementation at every meal, supplementation with HMB-FA, and studies conducted in Asia suggest that HMB possesses more beneficial effects (Table 4).

**Table 4:** Pooled estimates of the subgroup analyses.

Variable	Number of effect sizes	(I^2^)^1^	*P*-value	P_heterogenity_^2^	SMD (95%CI)^3^	P_-between_^4^
Cortisol						
Overall	12	84.1	0.14	<0.001	-0.39 (−0.92, 0.14)	
Intervention type						0.002
HMB-Ca	8	74.5	0.566	<0.001	−0.15 (−0.64, 0.35)	
HMB-FA	3	83.9	0.031	0.002	−1.82 (−3.46, −0.17)	
HMB	1	0	0.002	<0.001	0.80 (0.29, 1.31)	
Timing						0.343
Every meal	6	83.9	0.024	<0.001	−0.75 (−1.40, −0.10)	
Before training	3	74.5	0.142	0.020	−0.86 (−2.01, 0.29)	
After training	2	87.7	0.372	0.004	1.16 (−1.39, 3.71)	
Duration (weeks)						0.055
<8	5	92.5	0.056	<0.001	−1.39 (−2.82, 0.03)	
≥8	7	53.1	0.778	0.046	0.06 (−0.33, 0.44)	
Study design						0.186
Parallel	7	86.7	0.176	<0.001	−0.81 (−1.97, 0.36)	
Cross-over	5	70.7	0.883	0.009	0.03 (−0.39, 0.45)	
Location						0.005
Europe	8	65.7	0.352	0.005	0.19 (−0.21, 0.59)	
Asia	3	74.5	0.009	0.020	−1.61 (−2.82, −0.41)	
Testosterone						
Overall	9	72.0	0.001	<0.001	0.82 (0.35, 1.29)	
Intervention type						0.332
HMB-Ca	7	78.6	0.002	<0.001	0.92 (0.34, 1.49)	
HMB-FA	2	0	0.156	0.576	0.48 (−0.18, 1.15)	
Timing						0.024
Every meal	5	80.8	0.007	<0.001	0.84 (0.23, 1.45)	
Before training	2	0.0	0.680	0.553	0.14 (−0.52, 0.80)	
After training	2	0.0	<0.001	0.855	1.65 (0.77, 2.52)	
Duration (weeks)						0.723
<8	4	84.0	0.145	<0.001	0.86 (−0.30, 2.01)	
≥8	5	44.5	0.001	0.125	0.64 (0.25, 1.03)	
Study design						0.812
Parallel	5	47.8	0.023	0.105	0.76 (0.10, 1.42)	
Cross-over	4	85.6	0.016	<0.001	0.88 (0.17, 1.59)	
Location						0.622
Europe	5	44.5	0.001	0.125	0.64 (0.25, 1.03)	
Asia	3	87.4	0.185	<0.001	1.04 (−0.50, 2.57)	
IGF-1						
Overall	9	66.0	0.334	0.003	−0.18 (−0.54, 0.18)	
Intervention type						0.494
HMB-Ca	5	54.5	0.981	0.066	0.00 (−0.34, 0.35)	
HMB-FA	3	75.3	0.230	0.018	−0.69 (−1.82, 0.44)	
HMB	1	0.0	0.878	<0.001	0.06 (−0.66, 0.77)	
Timing						0.013
Every meal	4	0.0	0.259	0.471	0.12 (−0.09, 0.33)	
Before training	4	61.3	0.023	0.051	−0.83 (−1.55, −0.11)	
Duration (weeks)						0.982
<8	3	81.5	0.808	0.004	−0.15 (−1.39, 1.09)	
≥8	6	60.6	0.359	0.026	−0.17 (−0.53, 0.19)	
Location						0.435
Europe	4	69.8	0.375	0.019	−0.35 (−1.11, 0.42)	
Asia	3	0.0	0.915	0.565	−0.02 (−0.33, 0.29)	
Growth hormone						
Overall	6	78.9	0.910	<0.001	0.04 (−0.73, 0.82)	
Intervention type						0.396
HMB-Ca	3	0.0	0.214	0.408	−0.27 (−0.70, 0.16)	
HMB-FA	3	89.6	0.564	<0.001	0.53 (−1.28, 2.34)	
Timing						0.971
Every meal	2	0.0	0.592	0.387	0.14 (−0.38, 0.67)	
Before training	3	90.8	0.910	<0.001	0.11 (−1.77, 1.99)	
Duration (weeks)						0.017
<8	4	79.6	0.295	0.002	0.51 (−0.44, 1.45)	
≥8	2	0.0	0.007	0.657	−0.90 (−1.55, −0.24)	
Location						0.026
Europe	3	0.0	0.008	0.545	−0.68 (−1.18, −0.18)	
Asia	2	0.0	0.592	0.387	0.14 (−0.38, 0.67)	

SMD, standard mean difference; IGF-1, insulin-like growth factor 1.

1 Inconsistency, percentage of variation across studies due to heterogeneity.

2 Obtained from the Q-test.

3 Obtained from the fixed effects model.

4 Heterogeneity between groups.

#### 3.4.2 Findings from the meta-analysis of HMB and testosterone levels

The testosterone levels were measured in eight studies with nine treatment arms. According to the pooled results from the random-effects model, it was determined that testosterone levels were increased as the result of HMB supplementation (SMD: 0.82, 95% CI: 0.35, 1.29, *p* = 0.001) with significant heterogeneity between the studies (I^2^ = 72.0%, *p* < 0.001) (Figure 2B). As shown in Table 4, the timing of HMB supplementation is a source of heterogeneity. Studies conducted in Europe, with a duration of ≥8 weeks, using HMB-Ca, with time to take supplements at every meal or after training, indicated a significant increase in testosterone.

#### 3.4.3 Findings from the meta-analysis of HMB and IGF-1 levels

The levels of IGF-1 were measured in seven studies with nine treatments. According to the pooled estimates of the random-effects model, HMB supplementation had no significant effect on IGF-1 reduction (SMD: −0.18, 95% CI: −0.54, 0.18, *p* = 0.33), with significant heterogeneity between the studies (I^2^ = 66.0%, *p* = 0.003) (Figure 2C). It is possible that the timing of HMB supplementation could explain the heterogeneity between studies. Furthermore, supplementation with HMB before training resulted in a significant decrease in IGF-1 levels (Table 4).

#### 3.4.4 Findings from the meta-analysis of HMB and GH levels

Five studies with six treatment arms measured GH levels. As a result of the pooled estimates from the random-effects model, it was determined that GH levels did not increase significantly following HMB supplementation (SMD: 0.04, 95% CI: −0.73, 0.82, *p* = 0.91). Heterogeneity between the studies was significant (I^2^ = 78.9%, *p* < 0.001) (Figure 2D). In Table 4, heterogeneity could be attributed to the location of the studies as well as the duration of the interventions. A lower level of effects was observed in studies conducted in Europe and in those with a duration of ≥8 weeks (Table 4).

### 3.5 Sensitivity analysis

An analysis of sensitivity revealed that the effects of HMB supplementation on testosterone concentrations, IGF-1 levels, and GH levels were consistent across studies. However, the results for cortisol were specifically influenced by the study conducted by Fernández-Landa et al. (41) (Supplementary Figure S1).

### 3.6 Publication bias

The influence of HMB on the parameters of cortisol, testosterone, and IGF-1 hormones exhibited asymmetry in the funnel plots. In line with the “trim and fill” approach, it was inferred that two studies might have been missing for both potentially missing IGF-1 and testosterone (as depicted in Supplementary Figure S2). Furthermore, no significant publication bias among the studies was detected by either Begg’s rank correlation or Egger’s linear regression tests.

## 4 Discussion

This systematic review and meta-analysis, executed with strict adherence to the GRADE framework, provides a detailed perspective on the impact of HMB on key anabolic and catabolic hormones in adults. We hypothesized that HMB supplementation would significantly increase testosterone levels while having no impact on cortisol, IGF-1, or GH levels, which was partially confirmed. The findings demonstrated that HMB supplementation significantly increased testosterone levels, thereby supporting our hypothesis. However, consistent with our expectations, no significant changes were observed for cortisol, IGF-1, or GH levels, confirming the remainder of our hypothesis. This study collates the combined results of 15 CTs (involving 712 participants) to evaluate the effect of HMB and HMB-containing supplements on the hormonal responses in adults, both anabolic and catabolic.

Testosterone, a principal androgen, plays a crucial role in muscle protein synthesis, strength, and overall anabolic processes. Previous studies on HMB have shown mixed results regarding its effect on testosterone. While some earlier studies indicated no significant changes in testosterone levels with HMB supplementation (19, 20), others suggested potential increases similar to those found in this meta-analysis (23, 44). In 2020, Fernández-Landa et al. (41) conducted a 10-week RCT to evaluate the effects of HMB-free acid supplementation on strength, power, and hormonal adaptations following resistance training. This study suggested that HMB supplementation could have a synergistic effect on testosterone and the testosterone/cortisol ratio in the intervention group (41).

The discrepancies in the results can be ascribed to differences in study design, dosage levels, and characteristics of the participants. It is postulated that HMB may enhance testosterone levels through several potential mechanisms. One such hypothesis suggests that HMB could affect the hypothalamic–pituitary–gonadal (HPG) axis, thereby augmenting the secretion of luteinizing hormone (LH) from the pituitary gland. This hypothesis, in turn, stimulates the production of testosterone in the testes (45). Additionally, the role of HMB in reducing muscle damage and inflammation could indirectly create a more favorable environment for testosterone synthesis and action (46, 47). The analysis also indicated that HMB supplementation did not significantly reduce cortisol levels. Cortisol, a catabolic hormone, is involved in the stress response and can contribute to muscle protein breakdown (48). Maintaining stable cortisol levels is crucial for minimizing muscle degradation during periods of intense physical stress or training (49). The lack of a significant reduction in cortisol indicates that HMB’s anti-catabolic properties may not be linked to direct cortisol suppression (12). Earlier studies on HMB and cortisol levels have shown similar results, with most of them indicating no significant changes in cortisol with HMB supplementation (23, 50). Our findings on cortisol reduction align with those previously reported by Fernández-Landa et al. (51). This systematic review demonstrated that catabolic blood hormones (cortisol) did not show changes when the athletes were supplemented with creatine monohydrate plus HMB (51). The consistency observed across studies strengthens the conclusion that HMB does not exert its effects via cortisol modulation. Conversely, further studies are required to comprehend the impact of this supplementation on cortisol hormone responses (37). HMB may manifest its anti-catabolic effects through pathways that directly mitigate muscle protein breakdown at the cellular level, such as the inhibition of the Ub-pathway, rather than through systemic cortisol modulation (52). This finding highlights the potential of HMB to directly reduce muscle damage and protein degradation within muscle cells (53).

GH is another crucial anabolic hormone that plays a significant role in muscle growth, repair, and metabolism (54). Past investigations into the effects of HMB on GH have yielded inconsistent results. While some studies reported minor increases in GH, others found no effect (13, 38). This current meta-analysis consolidates these findings, offering more substantial evidence that HMB does not significantly alter GH levels. The absence of a significant increase in GH implies that the anabolic effects of HMB are not mediated through this pathway. This discovery is crucial as it outlines the specific hormonal mechanisms through which HMB may exert its effects, emphasizing that the increase in testosterone does not extend to GH modulation. The lack of impact of HMB on GH could be attributed to its primary mechanism involving muscle cell signaling pathways, such as the mammalian target of rapamycin (mTOR) pathway, which directly stimulates protein synthesis independently of GH (55, 56). This finding suggests that the anabolic effects of HMB might be more localized to muscle tissue than systemic endocrine alterations involving GH (20, 38).

IGF-1, a hormone with strong anabolic effects, is vital for muscle growth and repair (57). The current meta-analysis indicated that HMB supplementation did not significantly alter IGF-1 levels. Previous research on the impact of HMB on IGF-1 has been limited and inconclusive, with some studies reporting no significant changes (20, 21, 38). The findings of this meta-analysis align with these earlier observations, suggesting that HMB does not significantly alter IGF-1 levels (43). Recently, a systematic review and meta-analysis by Shakibaee et al. (22) found that HMB supplementation (3 g daily HMB for 7 weeks) led to greater adaptations in IGF-1 levels following a training intervention. It is important to note that this result was obtained by analyzing only two studies and considering the combined effects of resistance training and supplementation rather than supplementation alone. This finding indicates that IGF-1 concentrations have varied inconsistently during rest and resistance training in this population. IGF-1 works in tandem with GH to stimulate muscle hypertrophy and repair, and its stability in response to HMB suggests that the supplementation does not significantly influence the GH/IGF-1 axis (58, 59). The primary mechanism by which HMB may exert its anabolic effects is likely independent of the GH/IGF-1 axis (60). As mentioned, HMB has been shown to activate the mTOR pathway, a key regulator of protein synthesis and muscle growth, bypassing the need for IGF-1 modulation (61). This finding suggests that HMB can promote muscle anabolism directly at the muscle tissue level (62).

### 4.1 Recommendations for future research

The varied hormonal responses observed in this review stress the targeted effects of HMB supplementation. The significant increase in testosterone suggests a potential mechanism through which HMB can boost anabolic processes and support muscle hypertrophy and performance enhancements. However, the absence of significant changes in GH, cortisol, and IGF-1 indicates that the anabolic and anti-catabolic effects of HMB are not uniformly distributed across all major hormonal pathways involved in muscle metabolism. Future research should endeavor to clarify the exact mechanisms by which HMB influences testosterone levels and to investigate whether these effects result in long-term improvements in muscle mass, strength, and athletic performance. Furthermore, exploring the potential dose–response relationship and long-term safety of HMB supplementation will be crucial for formulating comprehensive guidelines for its use in various populations, including athletes, older adults, and those with muscle-wasting conditions (63, 64).

## 5 Strengths and limitations

To the best of our knowledge, this study represents the first systematic review and meta-analysis aimed at reviewing the role of HMB supplementation on hormonal changes (cortisol, testosterone, IGF-1, and GH) in adults across various clinical conditions. This systematic review and meta-analysis boasts several strengths, including a satisfactory number of studies. Moreover, the majority of the studies we included were deemed to be of high quality according to the Cochrane risk of bias tool. Given that the studies were conducted in various regions worldwide, our findings can be applied to adult populations globally. Furthermore, our search was not confined to a specific time or language.

However, it is important to acknowledge some limitations of this study. One of the primary limitations is the potential heterogeneity among the studies included in the systematic reviews and meta-analyses. Variations in study designs, populations, and intervention protocols might have influenced the observed results, making it challenging to draw definitive conclusions. Additionally, potential confounding factors, such as exercise and diet, could influence the hormonal response observed with HMB supplementation. These factors cannot be adequately controlled across all studies included in the systematic reviews and meta-analyses, making it difficult to attribute the observed effects solely to HMB. Furthermore, some of the included studies did not report the details of their randomization or the blinding of the outcome assessment process. Another limitation of this study is the significant heterogeneity in participant demographics, including age and sex, which complicates the generalizability of the findings. Finally, while our analysis focused on hormones central to metabolism (testosterone, cortisol, IGF-1, and GH), the lack of standardized reporting on other hormones, such as adrenaline and thyroid hormones, in existing studies limited our ability to explore the effects of HMB on the endocrine system in general. It is important to note that our geographic subgroup analyses were restricted to Asia and Europe, due to insufficient data from the Americas and the absence of studies from Africa. Future research should emphasize the importance of geographic diversity and comprehensive hormonal profiling, utilizing stratified analyses or focusing on more homogeneous populations, to maximize the generalizability of findings, fill existing gaps, and provide more clarity about the phenomena studied.

## 6 Conclusion

This research demonstrates that HMB supplementation leads to a statistically significant increase in testosterone levels. However, the magnitude of this change is small, and its physiological relevance remains unclear. Additionally, HMB does not significantly alter the GH, cortisol, or IGF-1 levels, suggesting that its anabolic and anti-catabolic effects are limited to specific hormonal pathways rather than being broadly distributed across muscle metabolism.

These findings highlight the need for a cautious interpretation of HMB’s potential benefits, particularly regarding its role in enhancing muscle hypertrophy or performance. Future studies should focus on exploring the mechanisms underlying HMB’s impact on testosterone and investigating its long-term effects and clinical implications. Special attention should be given to stratified analyses by sex and age, as well as the investigation of dose–response relationships. Future research should prioritize the development of functional food and beverage products that incorporate optimal doses of HMB. These products should be specifically formulated to address the unique needs of targeted consumer profiles, such as athletes and individuals who could benefit from mitigating muscle loss and inflammation. This targeted approach could enhance muscle health, improve exercise performance, and support overall wellbeing.

Research in homogeneous populations, such as athletes or individuals with muscle-wasting conditions, is essential to better understand the safety and efficacy of HMB supplementation. Comparative studies with other anabolic interventions could provide valuable insights, ultimately guiding evidence-based applications of HMB in clinical and sports settings.
