# Supplementary material for: β-Hydroxy-β-methyl butyrate (HMB) supplementation elevates testosterone levels without significant changes to cortisol, IGF-1, or growth hormone in adults: a GRADE-assessed systematic review and meta-analysis of controlled trials
Source: Front Nutr. 2025 Jun 19;12:1582135. doi: 10.3389/fnut.2025.1582135 (PMC12224445; doi:10.3389/fnut.2025.1582135)
Supplement: Supplementary file 1 [file Table_1.docx]

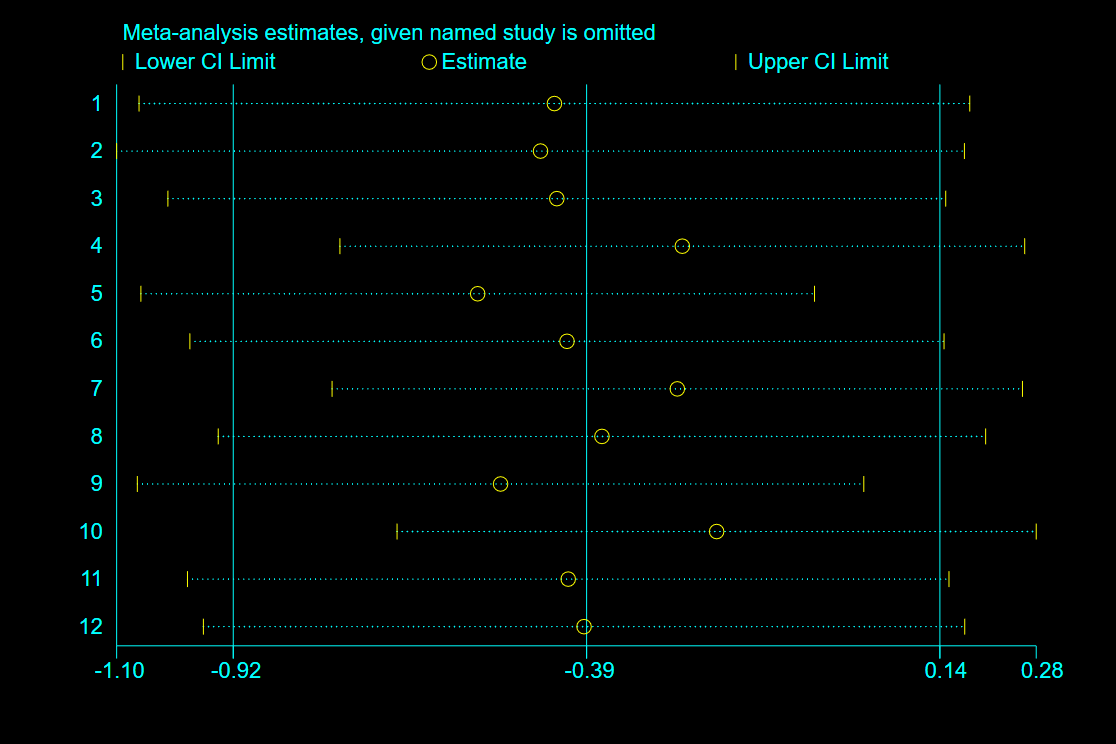


**(A)**


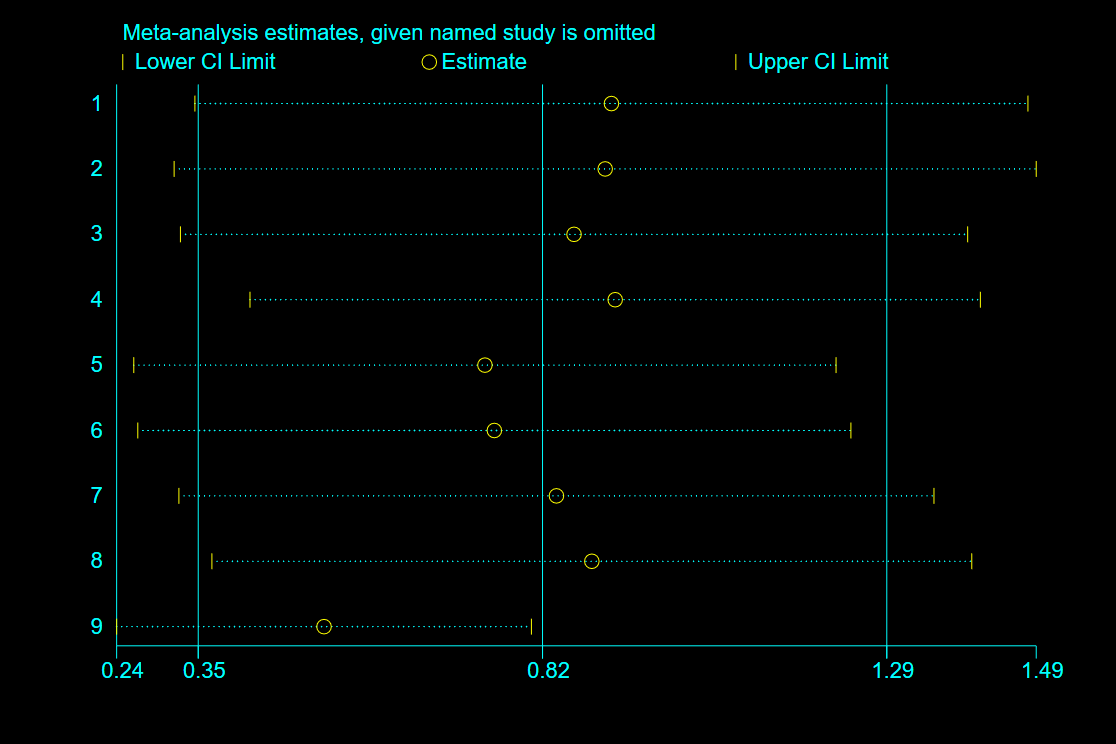


**(B)**


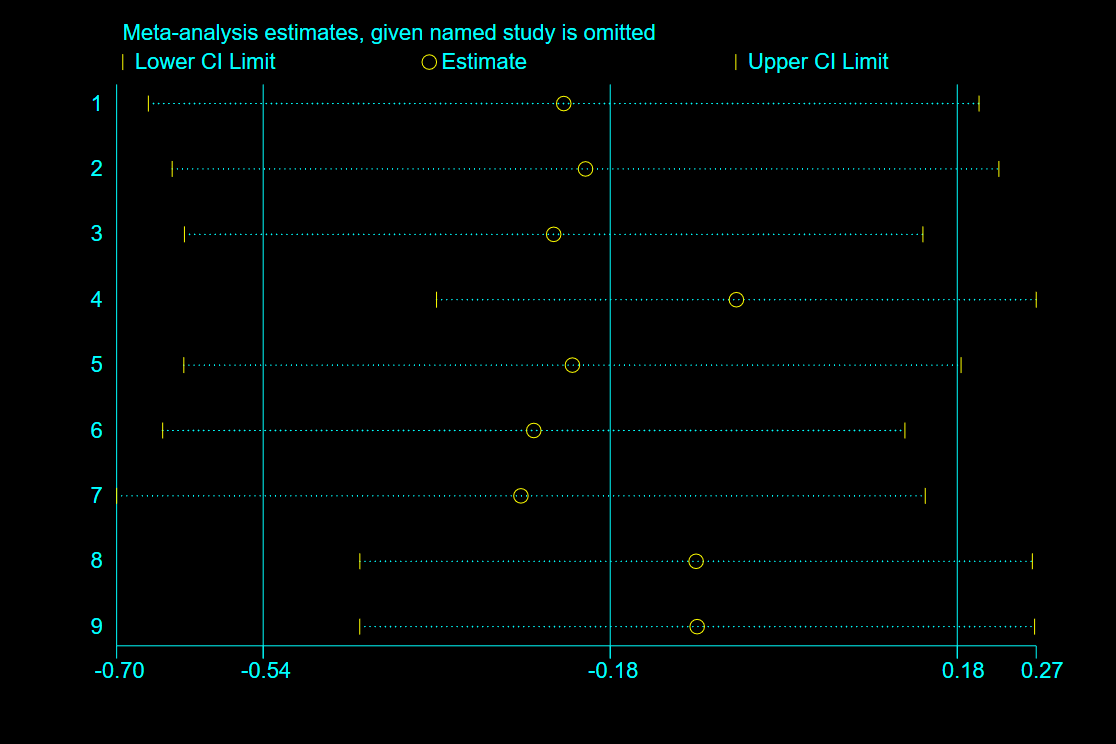


**(C)**


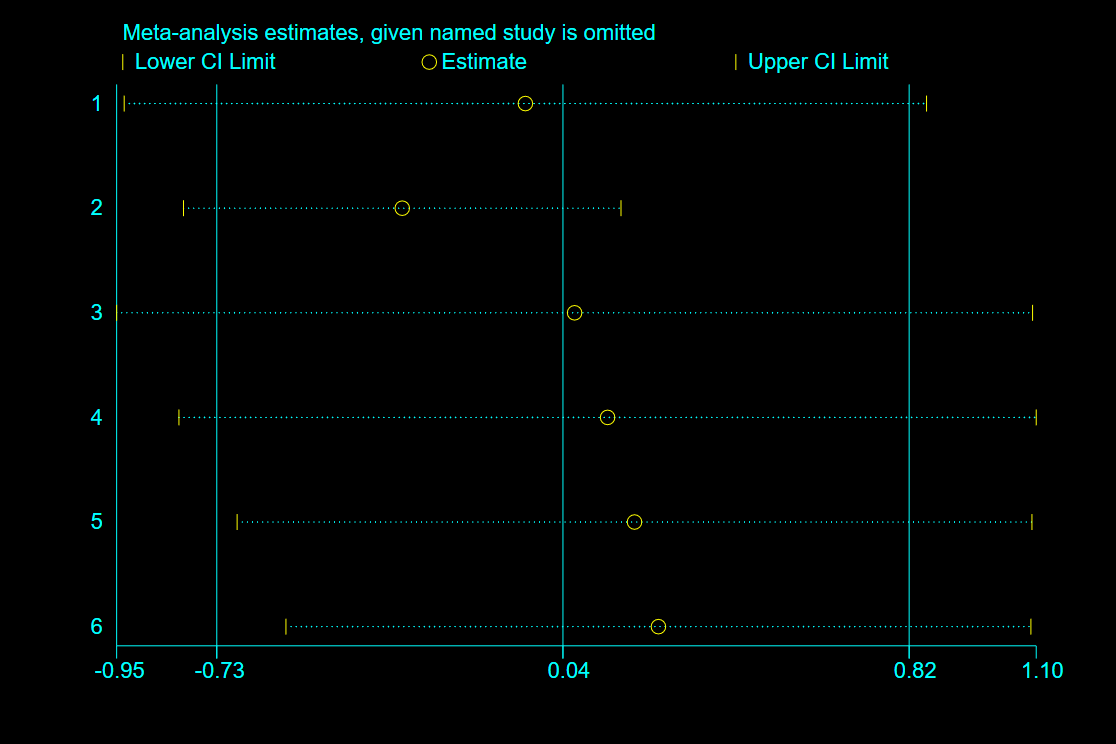


**(D)**

Fig. S1. A-D. The results of sensitivity analysis for SMD analysis of cortisol (A), testosterone (B), insulin-like growth factor 1 (C) and growth hormone (D).


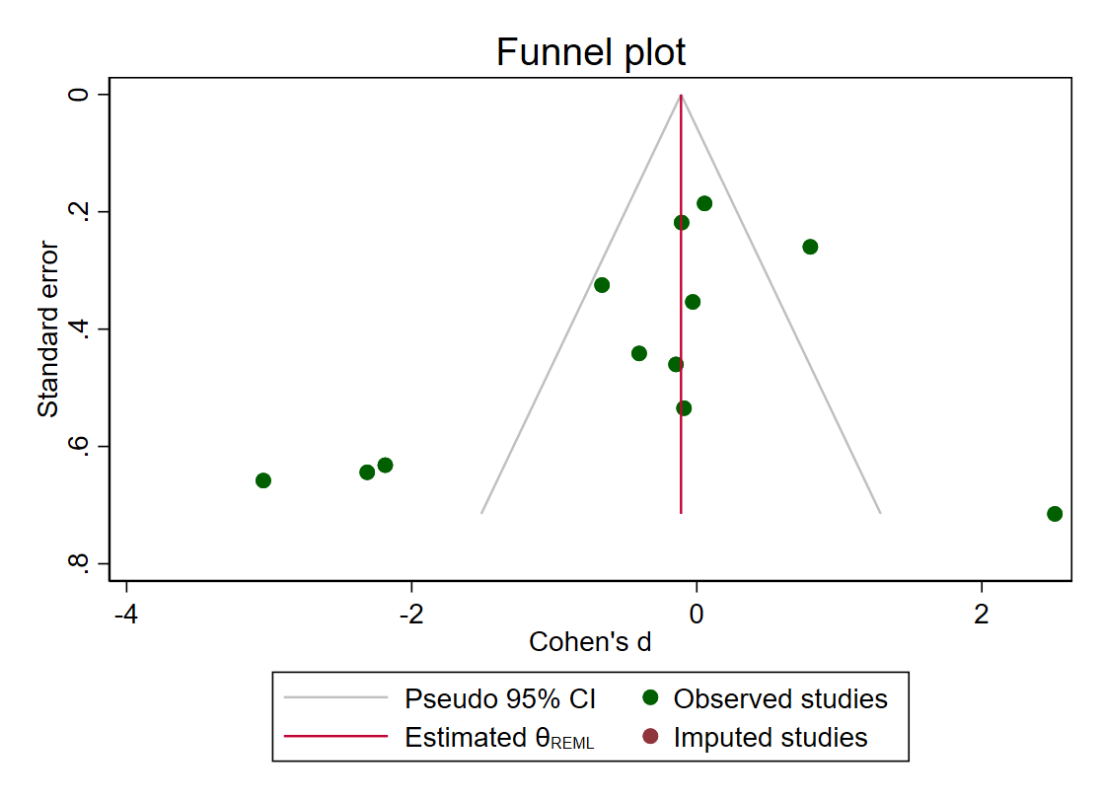


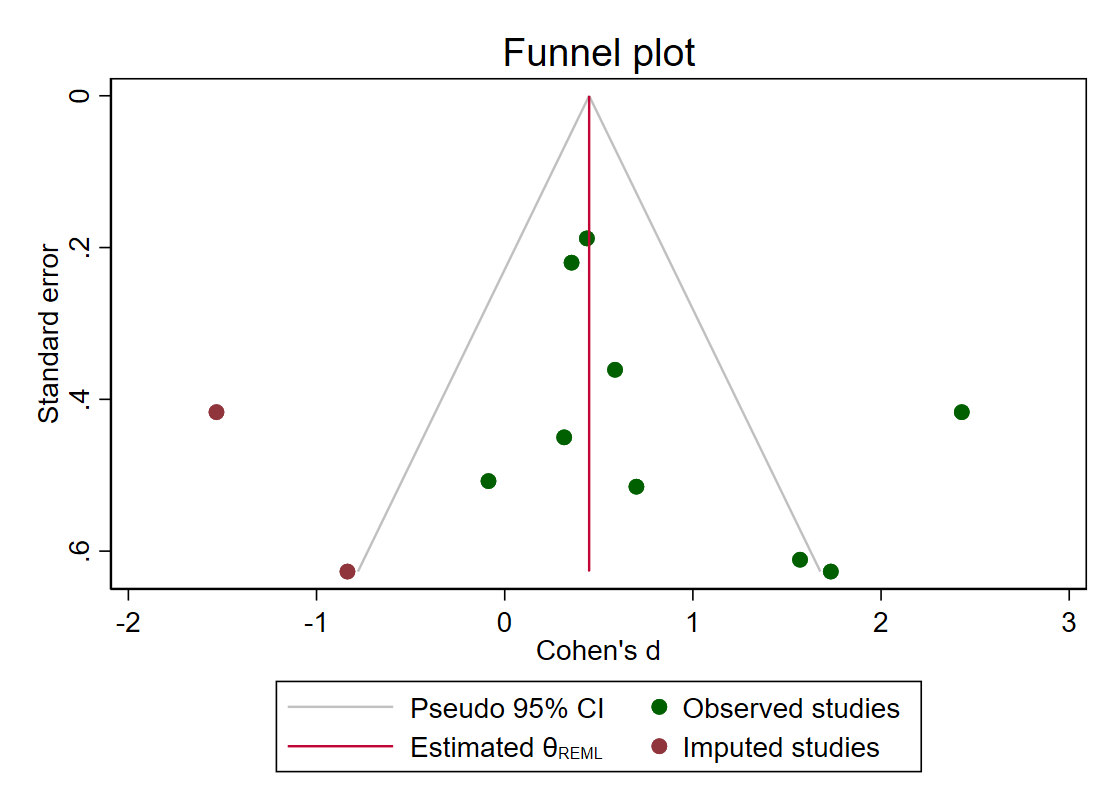


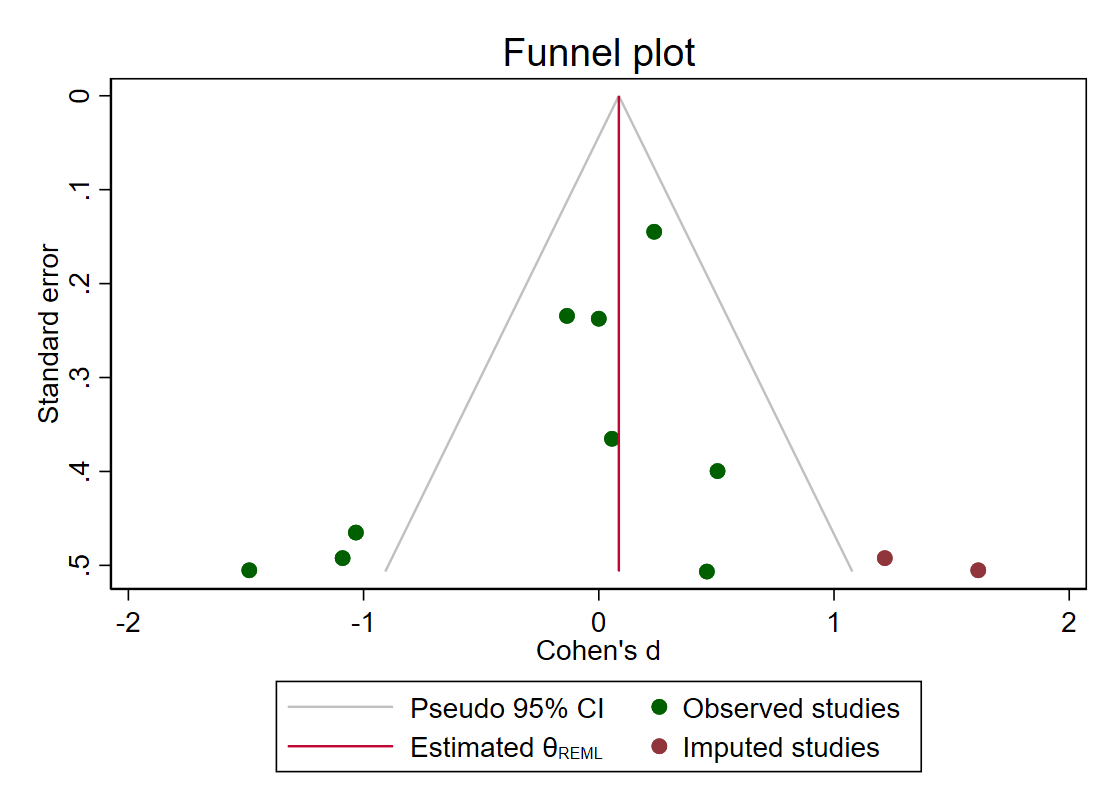


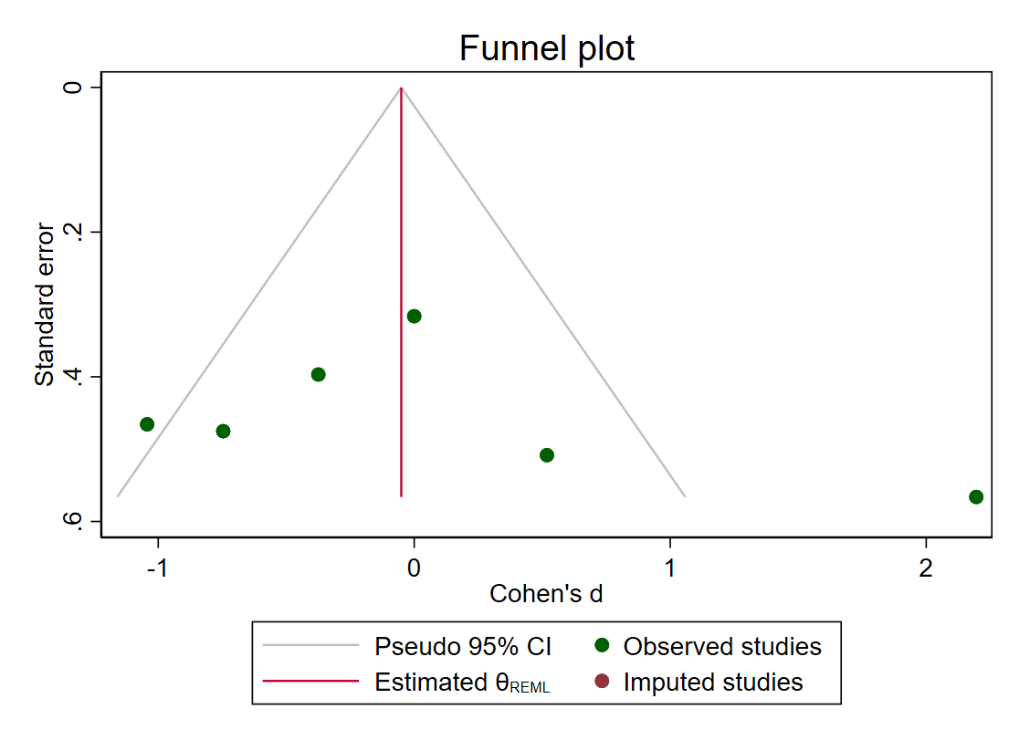


Fig. S2. A-D. Assessment of publication bias in the impact of β-Hydroxy-β-Methylbutyrate supplementation on catabolic and anabolic hormonal response in adults.

Table S1. Search strategy in databases.

| **Database** | **Strategy** | **Number** |
| --- | --- | --- |
| **PubMed** | (("beta-hydroxy beta-methylbutyrate") OR ("beta Hydroxy beta methylbutyric acid") OR ("hydroxy methylbutyrate") OR ("3-hydroxyisovaleric acid") OR ("b-hydroxybutyric acid") OR ("HMB") OR ("beta methylbutyrate") OR ("b-hydroxyb-methylbutyrate") OR ("sport supplements") OR ("hydroxy methylbutyrate") OR ("leucine metabolite") OR ("hmb fa") OR ("hmb ca") OR ("beta-hydroxy-beta-methylbutyrate free acid") OR ("beta-hydroxyisovaleric acid"[Supplementary Concept])) AND (("lipid profile") OR ("Total Cholesterol") OR ("HIGH DENSITY LIPOPROTEIN") OR ("Low-Density Lipoprotein") OR ("serum lipid") OR ("plasma lipid") OR ("plasma fatty acid") OR ("very low density lipoproteins") OR ("Apolipoproteins") OR ("Very-low density lipoprotein") OR ("Chylomicrons") OR ("triglyceride") OR ("Cholesterol") OR ("dyslipidemia") OR ("Cholesterol") OR "lipoproteins, hdl"[MeSH Terms] OR "cholesterol, ldl"[MeSH Terms] OR "Triglycerides"[MeSH Terms] OR "Apolipoproteins B"[MeSH Terms] OR "Fatty Acid-Binding Proteins"[MeSH Major Topic] OR "Fatty Acid-Binding Proteins"[MeSH Terms] OR "cholesterol, vldl"[MeSH Terms] OR "lipoproteins, vldl"[MeSH Terms] OR "lipoproteins, idl"[MeSH Terms] OR "Apolipoproteins"[MeSH Terms] OR "Apolipoproteins A"[MeSH Terms] OR "Chylomicrons"[MeSH Terms] OR "Lipids"[MeSH Terms] OR "Dyslipidemias"[MeSH Terms]) AND ("Randomized Controlled Trial" OR "Clinical Trial "OR "RCT" OR "Randomized Clinical Trial "OR Randomized OR Randomly OR Clinical OR Intervention OR Placebo OR Trial OR "Double blinded "OR "Double Blind Method" OR blind OR Control OR Random OR assignment OR "single blinded "OR "single-blind method" OR "Double-blind method "OR parallel OR "cross-over study") NOT ("in-vitro" OR "in-vivo" OR rabbit OR mouse OR rat OR mice OR cell OR monkey OR pig OR cow OR chicken OR goat OR sheep OR duck OR bacteria OR cat OR shrimp OR rooster OR warthogs OR broiler OR crab OR fowl) | **127** |
| **Scopus** | ( TITLE-ABS-KEY ( "beta-hydroxy beta-methylbutyrate" ) OR TITLE-ABS-KEY ( "&#946;-hydroxy &#946;-methylbutyrate" ) OR TITLE-ABS-KEY ( "beta Hydroxy beta methylbutyric acid" ) OR TITLE-ABS-KEY ( "hydroxy methylbutyrate" ) OR TITLE-ABS-KEY ( "3-hydroxyisovaleric acid" ) OR TITLE-ABS-KEY ( "b-hydroxybutyric acid" ) OR TITLE-ABS-KEY ( "HMB" ) OR TITLE-ABS-KEY ( "beta methylbutyrate" ) OR TITLE-ABS-KEY ( "b-hydroxyb-methylbutyrate" ) OR TITLE-ABS-KEY ( "sport supplements" ) OR TITLE-ABS-KEY ( "hydroxy methylbutyrate" ) OR TITLE-ABS-KEY ( "leucine metabolite" ) OR TITLE-ABS-KEY ( "HMB-FA" ) OR TITLE-ABS-KEY ( "HMB FA" ) OR TITLE-ABS-KEY ( "HMB-CA" ) OR TITLE-ABS-KEY ( "HMB CA" ) OR TITLE-ABS-KEY ( "beta-hydroxy-beta-methylbutyrate free acid" ) ) AND ( TITLE-ABS-KEY ( "lipid profile" ) OR TITLE-ABS-KEY ( "Total Cholesterol" ) OR TITLE-ABS-KEY ( "TC" ) OR TITLE-ABS-KEY ( "HIGH DENSITY LIPOPROTEIN" ) OR TITLE-ABS-KEY ( "HDL" ) OR TITLE-ABS-KEY ( "Low-Density Lipoprotein" ) OR TITLE-ABS-KEY ( "LDL" ) OR TITLE-ABS-KEY ( "TG" ) OR TITLE-ABS-KEY ( "apo B" ) OR TITLE-ABS-KEY ( "serum lipid" ) OR TITLE-ABS-KEY ( "plasma lipid" ) OR TITLE-ABS-KEY ( "plasma fatty acid" ) OR TITLE-ABS-KEY ( "very low density lipoproteins" ) OR TITLE-ABS-KEY ( "apolipoproteins" ) OR TITLE-ABS-KEY ( "Apo A" ) OR TITLE-ABS-KEY ( "VLDL" ) OR TITLE-ABS-KEY ( "Very-low density lipoprotein" ) OR TITLE-ABS-KEY ( "chylomicrons" ) OR TITLE-ABS-KEY ( "triglyceride" ) OR TITLE-ABS-KEY ( "lipids" ) OR TITLE-ABS-KEY ( "cholesterol" ) OR TITLE-ABS-KEY ( "dyslipidemia" ) ) AND ( TITLE-ABS-KEY ( "Randomized Controlled Trial" ) OR TITLE-ABS-KEY ( "Clinical Trial" ) OR TITLE-ABS-KEY ( "RCT" ) OR TITLE-ABS-KEY ( "Randomized Clinical Trial" ) OR TITLE-ABS-KEY ( randomized ) OR TITLE-ABS-KEY ( randomly ) OR TITLE-ABS-KEY ( clinical ) OR TITLE-ABS-KEY ( intervention ) OR TITLE-ABS-KEY ( placebo ) OR TITLE-ABS-KEY ( trial ) OR TITLE-ABS-KEY ( "Double blinded" ) OR TITLE-ABS-KEY ( "Double Blind Method" ) OR TITLE-ABS-KEY ( blind ) OR TITLE-ABS-KEY ( control ) OR TITLE-ABS-KEY ( random ) OR TITLE-ABS-KEY ( assignment ) OR TITLE-ABS-KEY ( "single blinded" ) OR TITLE-ABS-KEY ( "single-blind method" ) OR TITLE-ABS-KEY ( "Double-blind method" ) OR TITLE-ABS-KEY ( parallel ) OR TITLE-ABS-KEY ( "cross-over study" ) AND NOT TITLE-ABS-KEY ( "in-vitro" ) OR TITLE-ABS-KEY ( "in-vivo" ) OR TITLE-ABS-KEY ( rabbit ) OR TITLE-ABS-KEY ( mouse ) OR TITLE-ABS-KEY ( rat ) OR TITLE-ABS-KEY ( mice ) OR TITLE-ABS-KEY ( cell ) OR TITLE-ABS-KEY ( monkey ) OR TITLE-ABS-KEY ( pig ) OR TITLE-ABS-KEY ( cow ) OR TITLE-ABS-KEY ( chicken ) OR TITLE-ABS-KEY ( goat ) OR TITLE-ABS-KEY ( sheep ) OR TITLE-ABS-KEY ( duck ) OR TITLE-ABS-KEY ( bacteria ) OR TITLE-ABS-KEY ( cat ) OR TITLE-ABS-KEY ( shrimp ) OR TITLE-ABS-KEY ( rooster ) OR TITLE-ABS-KEY ( warthogs ) OR TITLE-ABS-KEY ( broiler ) OR TITLE-ABS-KEY ( crab ) OR TITLE-ABS-KEY ( fowl ) ) | **268** |
| **Web of Science** | (((ALL=("beta-hydroxy beta-methylbutyrate" OR "β-hydroxy β-methylbutyrate" OR "beta Hydroxy beta methylbutyric acid" OR "hydroxy methylbutyrate" OR "3-hydroxyisovaleric acid" OR "b-hydroxybutyric acid" OR "HMB" OR "beta methylbutyrate" OR "b-hydroxyb-methylbutyrate" OR "sport supplements" OR "hydroxy methylbutyrate" OR "hydroxy methylbutyrate" OR "leucine metabolite" OR "HMB-FA" OR "HMB FA" OR "HMB-CA" OR "HMB CA" OR "beta-hydroxy-beta-methylbutyrate free acid")) AND ALL=("lipid profile" OR "Total Cholesterol" OR "TC" OR "HIGH DENSITY LIPOPROTEIN" OR "HDL" OR "Low-Density Lipoprotein" OR "LDL" OR "TG" OR "apo B" OR "serum lipid" OR "plasma lipid" OR "plasma fatty acid" OR "very low density lipoproteins" OR "apolipoproteins" OR "Apo A" OR "VLDL" OR "Very-low density lipoprotein" OR "chylomicrons " OR "lipids " OR "triglyceride" OR "cholesterol" OR "dyslipidemia")) AND ALL=("Randomized Controlled Trial" OR "Clinical Trial" OR "RCT" OR "Randomized Clinical Trial" OR "Randomized" OR "Randomly" OR "Clinical" OR "Intervention" OR "Placebo" OR "Trial" OR "Double blinded" OR "Double Blind Method" OR "blind" OR "Control" OR "Random" OR "assignment" OR "single blinded" OR "single-blind method" OR "Double-blind method" OR "parallel" OR "cross-over study")) NOT ALL=("in-vitro" OR "in-vivo" OR "rabbit" OR "mouse" OR "chicken" OR "duck" OR "mice" OR "rat" OR "monkey" OR "pig" OR "cell" OR "cow" OR "goat" OR "bacteria" OR "sheep" OR "cat" OR "shrimp" OR "rooster" OR "warthogs" OR "broiler" OR "crab" OR "fowl") | **164** |

Duplicate: 79 items

Table S2. GRADE profile of HMB supplementation on hormones.

| **Certainty assessment** | | | | | | | **№ of patients** | | **Effect** | **Certainty** | **Importance** |
| --- | --- | --- | --- | --- | --- | --- | --- | --- | --- | --- | --- |
| **№ of studies** | **Study design** | **Risk of bias** | **Inconsistency** | **Indirectness** | **Imprecision** | **Other considerations** | **[intervention]** | **[comparison]** | **Absolute (95% CI)** |  |  |
| **Cortisol** | | | | | | | | | | | |
| 10 | randomised trials | not serious | serious^a^ | not serious | serious^b^ | none | 232 | 225 | SMD **0.39 SD lower** (0.92 lower to 0.14 higher) | ⨁⨁◯◯ Low | IMPORTANT |
| **Testosterone** | | | | | | | | | | | |
| 8 | randomised trials | not serious | not serious^c^ | not serious | not serious | none | 179 | 174 | SMD **0.82 SD higher** (0.35 higher to 1.29 higher) | ⨁⨁⨁⨁ High | IMPORTANT |
| **Igf 1** | | | | | | | | | | | |
| 7 | randomised trials | not serious | not serious^d^ | not serious | serious^e^ | none | 229 | 240 | SMD **0.18 SD lower** (0.54 lower to 0.18 higher) | ⨁⨁⨁◯ Moderate | IMPORTANT |
| **GH** | | | | | | | | | | | |
| 5 | randomised trials | not serious | not serious^f^ | not serious | serious^g^ | none | 72 | 62 | SMD **0.04 SD higher** (0.73 lower to 0.82 higher) | ⨁⨁⨁◯ Moderate | IMPORTANT |

**CI:** Confidence interval; **SMD:** Standardised mean difference

#### Explanations:

a. Serious inconsistency since I2 = 69.5%. Downgraded.

b. Serious imprecision since combined results from the random-effects model showed a non-significant reduction in cortisol following HMB supplementation wasn’t significant (p = 0.14). Downgraded.

c. Serious inconsistency since I2 = 72.0 %. However, the value of was I2 <50% in the subgroup of trials with duration ≥8 weeks and the significance, direction, and magnitude of the effect remained unchanged (SMD: 0.64, 95% Cl: 0.25, 1.03; n=5, I2 = 44.5%). Not downgraded.

d. Serious inconsistency since I2 = 66.0 %. However, the value of was I2 <50% in the subgroup of trials conducted in Asia and the significance, direction, and magnitude of the effect remained unchanged (SMD: -0.02, 95% Cl: -0.33, 0.29; n=4, I2 = 0 %). Not downgraded.

e. Serious imprecision since combined results from the random-effects model showed a non-significant reduction in Igf 1 following HMB supplementation wasn’t significant (p = 0.33). Downgraded.

f. Serious inconsistency since I2 = 78.9 %. However, the value of was I2 <50% in the subgroup of trials conducted in Asia and the significance, direction, and magnitude of the effect remained unchanged (SMD: 0.14, 95% Cl: -0.38, 0.67; n=3, I2 = 0%). Not downgraded.

g. Serious imprecision since combined results from the random-effects model showed a non-significant reduction in GH following HMB supplementation wasn’t significant (p = 0.91). Downgraded.
